# Carcinogenetic initiation contributed by EpCAM+ cancer cells in orthotopic HCC models of immunocompetent and athymic mice

**DOI:** 10.18632/oncotarget.27454

**Published:** 2020-06-02

**Authors:** Harshul Pandit, Yan Li, Qianqian Zheng, Wei Guo, Youxi Yu, Suping Li, Robert C.G. Martin

**Affiliations:** ^1^Division of Surgical Oncology, Hiram C. Polk Jr. M.D. Department of Surgery, University of Louisville School of Medicine, Louisville, KY 40202, USA; ^2^Department of Pathophysiology, Basic Medicine College, China Medical University, Shenyang 110122, China; ^3^Department of Hematology, The First Hospital of Jilin University, Changchun 130021, China; ^4^Department of Hepatobiliary and Pancreatic Surgery, The First Hospital of Jilin University, Changchun 130021, China; ^5^Department of Pharmacology and Toxicology, University of Louisville School of Medicine, Louisville, KY 40202, USA

**Keywords:** hepatocellular carcinoma, Wnt/β-catenin signaling, cancer stem cells, epithelial cell adhesion molecule (EpCAM), tumor-initiating cells

## Abstract

Purpose: Overexpression of epithelial cell adhesion molecule (EpCAM) correlates with poor prognosis, therapeutic failure and early tumor recurrence in hepatocellular carcinoma (HCC) patients. The tumor microenvironment dictates the fate of tumor-initiating cancer stem cells (CSCs); however, very limited studies were attempted to evaluate CSC tumorigenesis in the liver microenvironment. Here, we have systemically investigated the role of EpCAM+ cancer cells in tumor initiation in orthotopic HCC models.

Results: Control mice and the mice with bland steatosis failed to develop tumors. In the mice with steatohepatitis, EpCAM+ CSCs have shown significantly increased ability in terms of tumor initiation and growth, compared to that with EpCAM- non-CSCs inoculation (*p* < 0.005). For Hep3B inoculation, EpCAM-High group has shown significantly higher tumor growth compared with EpCAM-Low (*p* < 0.005). For HepG2 inoculation, both EpCAM-High and EpCAM-Low groups confirmed similar tumor incidence and growth.

Methods: Diet-induced compromised microenvironments were established to mimic clinical fatty liver and non-alcoholic steatohepatitis (NASH) patients and the tumorigenic capabilities of Hepa1-6 cells were evaluated. CSCs were enriched by spheroid culture and labeled with copGFP for EpCAM+ CSCs and with mCherry for non-CSCs. FACS-sorted cells were inoculated into left liver lobes, and tumor growth was monitored by high-frequency ultrasound. The subpopulations of Hep3B and HepG2 cells in terms of EpCAM-Low and EpCAM-High were evaluated in the orthotopic model of athymic mice.

Conclusions: NASH microenvironment promotes the EpCAM+ CSCs initiated tumorigenesis in immunocompetent mouse model. Differential EpCAM expression demonstrates distinct tumor biology in athymic mouse models.

## INTRODUCTION

Hepatocellular carcinoma (HCC), a type of epithelial cancer, is the most common primary liver cancer (80–90%) in the United States [1]. HCC is the fifth most common cancer in men and seventh in women, and accounts for the third major cause of cancer-related deaths worldwide [2, 3]. HCC confers the highest death rate (~2.4%) among all cancers in United States [4]. A subset of cancer cells within the tumor microenvironment, defined as cancer stem cells (CSCs), contributes to aggressive tumor initiation, therapeutic treatment resistance, and tumor relapse in HCC patients [5].

The CSC concept has been elucidated in regard to tumor heterogeneity within primary HCC, and it helps to understand therapeutic resistance and early relapse [5–7]. Because of vast heterogeneity, no single marker can define the CSCs exclusively [8]. Identification of CSCs from human HCC tumors and human HCC cell lines has been performed using various CSC surface biomarkers, including CD90+, CD44+, epithelial cell adhesion molecule (EpCAM+), CD133+, OV6+, aldehyde dehydrogenase 1 (ALDH1+), and alpha-fetoprotein (AFP+) [5, 7, 9, 10]. EpCAM, one of the most characterized and well-accepted CSC markers, is associated with poor prognosis in HCC patients [10–14]. EpCAM is a target of Wnt/β-catenin signaling, and inhibiting Wnt/β-catenin signaling has been shown to destroy the EpCAM+ cells [13, 15]. There is no previous study to investigate EpCAM-expressing CSCs for their tumorigenic ability in a compromised liver microenvironment such as non-alcoholic fatty liver disease (NAFLD).

Increased incidence of HCC has been attributed to an increased incidence of non-alcoholic fatty liver disease [16]. At least 25% of the US population has NAFLD, and NAFLD is becoming an epidemic [17, 18]. NASH is a severe form of NAFLD and present in 6% to 17% population [18]. Approximately 50% cases of NASH progress to HCC without cirrhosis and confer a new challenge to clinicians and researchers to understand NASH-mediated HCC transition [19]. Significant gaps remain to investigate the mechanisms by which NAFLD/NASH and its metabolic risk factors promote HCC carcinogenetic transformation. No previous study has been done to evaluate the CSC tumorigenesis in the liver microenvironment with NASH in immunocompetent mouse models.

Previously, we have demonstrated that an EpCAM+ CSC subset was significantly enriched in heterogeneous Hepa1-6 CSC spheroids, and these CSC spheroids acquired high tumorigenic potential in an orthotopic immunocompetent C57L/J mouse model [20]. In this study, for the first time, we evaluate EpCAM+ CSCs from 3 different HCC cell lines in immunocompetent and immunocompromised mouse models via orthotopic inoculation. We studied the tumorigenesis fate of EpCAM+ Hepa1-6 cells in 3 different liver microenvironments (normal, bland steatosis, and steatohepatitis) using an immunocompetent C57L/J mouse model. We also investigate the tumorigenesis fate of EpCAM-High and EpCAM-Low subsets from two EpCAM positive human cell lines, i. e. Hep3B and HepG2, using orthotopic athymic Nu/J mouse model.

## RESULTS

### EpCAM and β-catenin overexpressioin in HCC patients

**Figure 1:**
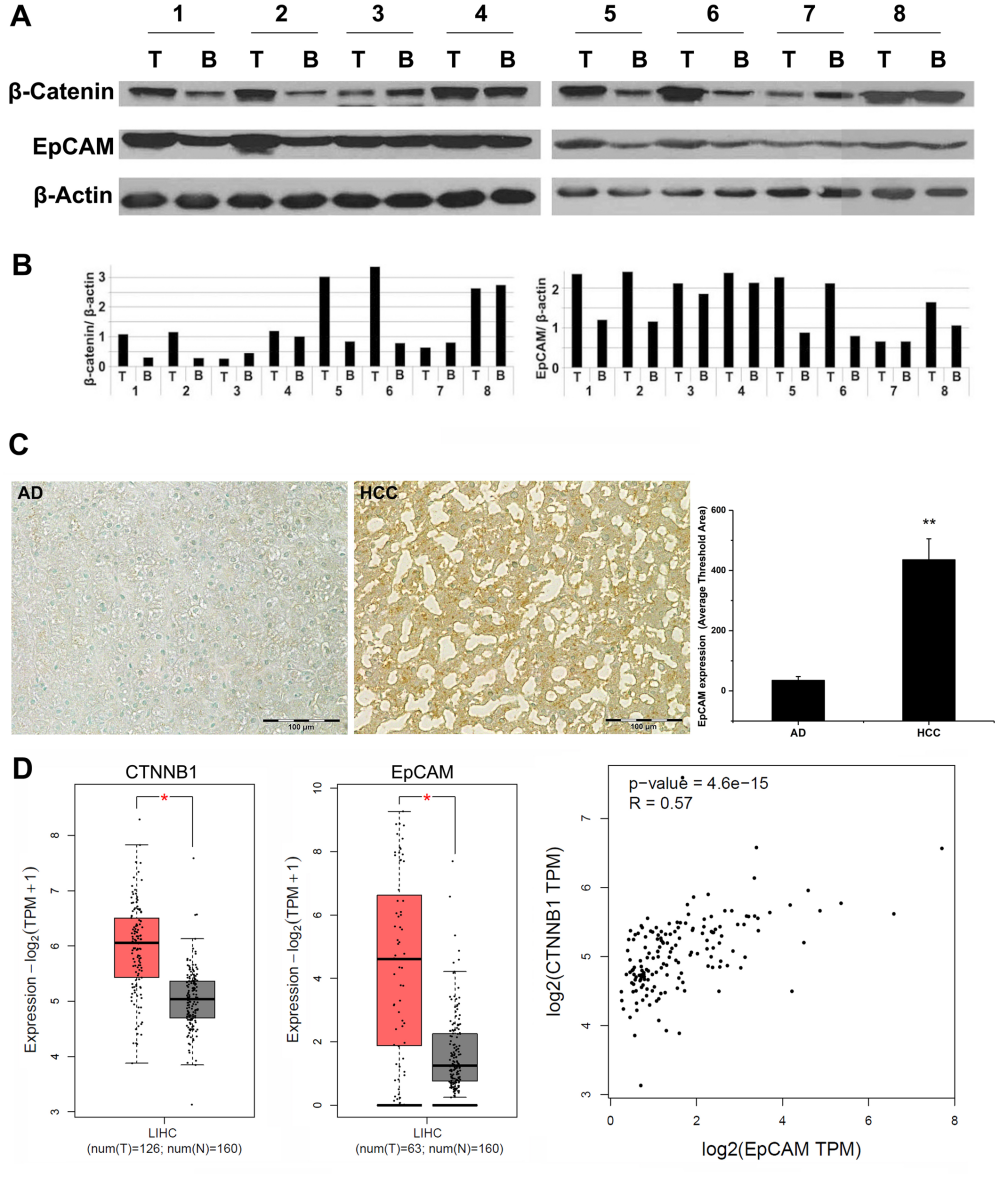
Analysis of human HCC specimens. Total proteins were extracted from 8 pairs of human HCC specimens (tumor tissue and adjacent benign tissue) and analyzed by SDS-PAGE followed by Western blot for EpCAM and β-catenin expression. (**A**) Western blot. (**B**) densitometry image analysis of EpCAM (left) and β-catenin (right) expression in malignant tumor tissue and adjacent benign tissue (*n* = 8). T: tumor tissue; B: adjacent benign tissue. A total of 24 pairs of HCC specimens were analyzed by IHC staining. (**C**) Left: Representative images of IHC EpCAM staining of human tissue specimens. Right: Quantified EpCAM expression (*n* = 24, paired benign AD and HCC specimens). 20× magnification (Bar = 100 µm) AD: adjacent normal liver; HCC: hepatocellular carcinoma. (**D**) Gene Expression Profiling Interactive Analysis (GEPIA) was used to provide key interactions and functions based on the Cancer Genome Atlas (TCGA) and Genotype-Tissue Expression (GTEx) dataset for transcriptomic analysis (http://gepia.cancer-pku.cn) [PMID: 28407145]. The Spearman method was used to determine the correlation coefficient. The cBioPortal for Cancer Genomics (http://cbioportal.org) provides a Web resource for exploring, visualizing, and analyzing multidimensional cancer genomics data. ^*^ *p* < 0.01.

Total protein was extracted from malignant tissues as well as the paired adjacent benign tissues from 24 HCC patients, while the protein levels of β-catenin and EpCAM were evaluated by Western blot from 8 HCC patients. As shown in Figure 1A and 1B, concomitant high levels of EpCAM and β-catenin were also detected in malignant tissues, compared to the benign adjacent tissues. A total 3 of 8 patients showed either concomitant decreased β-catenin and EpCAM or no change, compared with adjacent benign control. Overall, Western blot analysis confirmed that EpCAM and β-catenin expression were positively correlated in the HCC patient samples. We further examined 24 pairs of HCC tissues and adjacent benign control by IHC (Figure 1C). The results showed a significant increase in EpCAM expression in malignant specimens of HCC (483.75 ± 119.92, *p* < 0.0001) when compared with the adjacent benign liver tissue within the resected specimens (38.44 ± 7.31). Patients with increase EpCAM expression did demonstrate a more aggressive phenotype with a infiltrative pattern and high incidence of micro-vascular invasion and poor differentiation when compared to lower expressing EpCAM tumors. The numbers were too small to perform any type of comparsion at this time. A web-based database, Gene Expression Profiling Interactive Analysis (GEPIA) was used to further confirm our results of HCC human samples. The results indicated that the high expressions of both β-catenin and EpCAM were significantly increased in the HCC tissues compared to the adjacent benign tissues. By co-expression analysis, there was a positive correlation between β-catenin and EpCAM expression in HCC patients (R = 0.57, *P* < 0.001) (Figure 1D). Analysis of TCGA database revealed that CTNNB1 (β-catenin gene) is the third most mutated gene in liver cancer patients (24% of cases in cohort, Supplementary Figure 1). EpCAM, a well-reported CSC marker, is a downstream target of CTNNB1 (β-catenin).

### NASH microenvironment favors the EpCAM+ Hepa1-6 CSCs to initiate HCC in orthotopic immunocompetent mouse models

**Figure 2:**
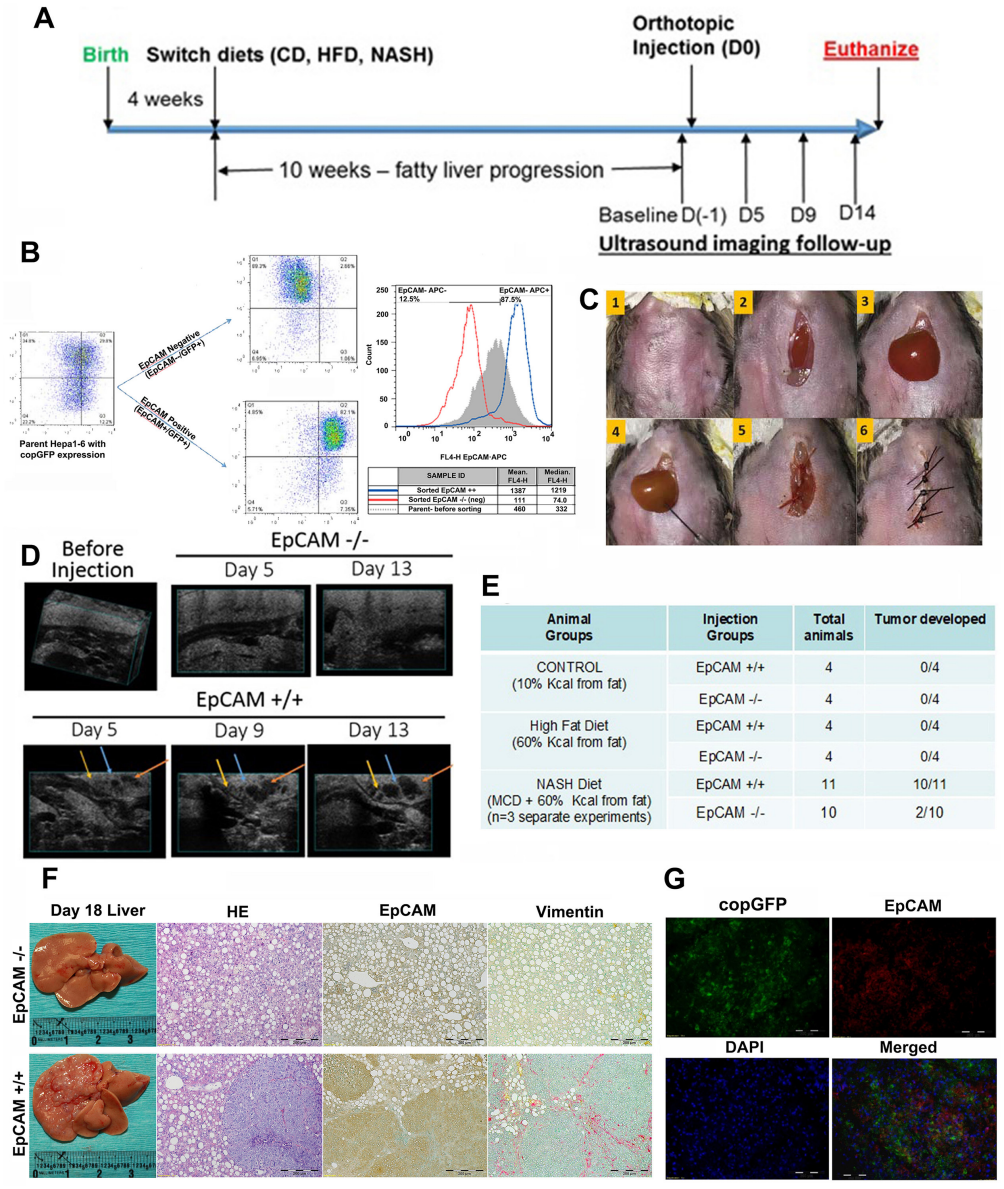
NASH microenvironment is tumorigenic for EpCAM+ Hepa1-6 CSCs in orthotopic immunocompetent mouse models. (**A**) Simplified experimental workflow. (**B**) Lentivirus-transfected Hepa1-6 cells expressing copGFP were sorted by flow cytometry for EpCAM−/− and EpCAM+/+ populations. (**C**) Representative step-by-step surgical procedure – 2 million (2 × 10^6) sorted cells were injected into the left liver lobes of C57L/J diet induced control, HFD, or NASH mice by survival surgery. (**D**) Non-invasive ultrasound images recorded at different times to track tumor progression in EpCAM−/− and EpCAM +/+ groups of NASH liver animals. EpCAM+ group NASH animals were confirmed with tumor growth at D5 time-point. Representative images showing tracking of each tumor nodule growth on D5, D9 and D13. (**E**) Experimental outcomes of orthotopic injection of EpCAM-expressing CSCs in different liver microenvironments showed that only the NASH liver microenvironment was favorable to EpCAM+ CSCs. No tumor growth was detected in control and HFD groups; (**F**) Representative gross liver pictures of euthanized mice on D18, and representative IHC-P staining, H&E stain, EpCAM, Vimentin (from left to right). EpCAM and Vimentin were significantly higher in tumors of the EpCAM+ group in NASH animals. (**G**) IHC-Fz staining of tumor from EpCAM+ CSCs in orthotopic NASH mouse model. Green fluorescence was natural fluorescence from copGFP. EpCAM was detected by Rabbit-Anti-EpCAM (1:100) followed by secondary signal amplification using Goat-Anti-Rabbit (1:500, Alexafluor – 594). FITC channel was used for detecting copGFP and TXRED channel used for detecting EpCAM. DAPI stain for nuclei was detected by UV channel filter. The individual images of copGFP and EpCAM as well as the merged images confirmed copGFP and EpCAM co-expression.

We have successfully established diet-induced mouse models to represent distinct liver microenvironments (Supplementary Figures 2 and 3), with HFD-feeding and CDAHFD-feeding for steatosis and steatohepatitis. Figure 2A showed a simplified experimental workflow. A total of 2 million (2 × 10^6) sorted EpCAM+(positive) or EpCAM- (negative) subpopulations of Hepa1-6 cells labeled with copGFP (Figure 2B) were orthotopically injected into left liver lobes of animals by survival surgery (Figure 2C). Animals were followed up by ultrasound imaging (Figure 2E) to track tumor growth. As shown by ultrasound, neither control-diet feeding nor HFD feeding animals showed any tumor nodule at day 13 (Figure 2D), indicating that both EpCAM+ and EpCAM- Hepa1-6 cells failed to initiate tumor in healthy liver microenvironment and bland steatosis. In CDAHFD-feeding group, the animals with EpCAM+ CSC injection had detectable tumor initiation as early as day 5. On day 13, the ultrasound-identified tumors had grown larger. There was no tumor detected by ultrasound in the EpCAM-cell injection group of animals with CDAHFD-feeding. All the animals were euthanized on Day 18. Macroscopic liver anatomy and dissection confirmed the ultrasound-identified tumors. Inoculation with EpCAM+ cells in the CDAHFD-feeding group had initiated tumors in almost all animals (*n* = 10/11 animals confirmed tumor, *n* = 3 independent experiments with at least *n* = 3 animals/group/attempt). Inoculation with EpCAM- cells in the CDAHFD-feeding had initiated tumors in 2 mice (*n* = 2/10) (Figure 2E). Pathological analysis by H&E staining confirmed macroscopic findings of HCC (Figure 2F). IHC-P analysis showed significantly increased EpCAM expression in the hepatic tissue compared to no-tumor EpCAM- animals (Figure 2F). IHC-P analysis further found increased expression of vimentin in EpCAM+ NASH tumors (Figure 2F). As the inoculation of copGFP-expressing stable Hepa1-6 cells was for the purpose of lineage tracking of tumor being initiated from injected cells, we further performed immunofluorescence analysis in frozen section (IHC-Fz) of tumor tissue (Figure 2G), and the result confirmed that the co-expression of EpCAM and copGFP. These findings suggested that EpCAM+ CSC-initiated tumorigenesis is favored in NASH microenvironments of immunocompetent mice, compared to those with normal liver or bland steatosis.

### Tumor growth in EpCAM+ NASH animals displayed aggressive form of HCC tumorigenesis

**Figure 3:**
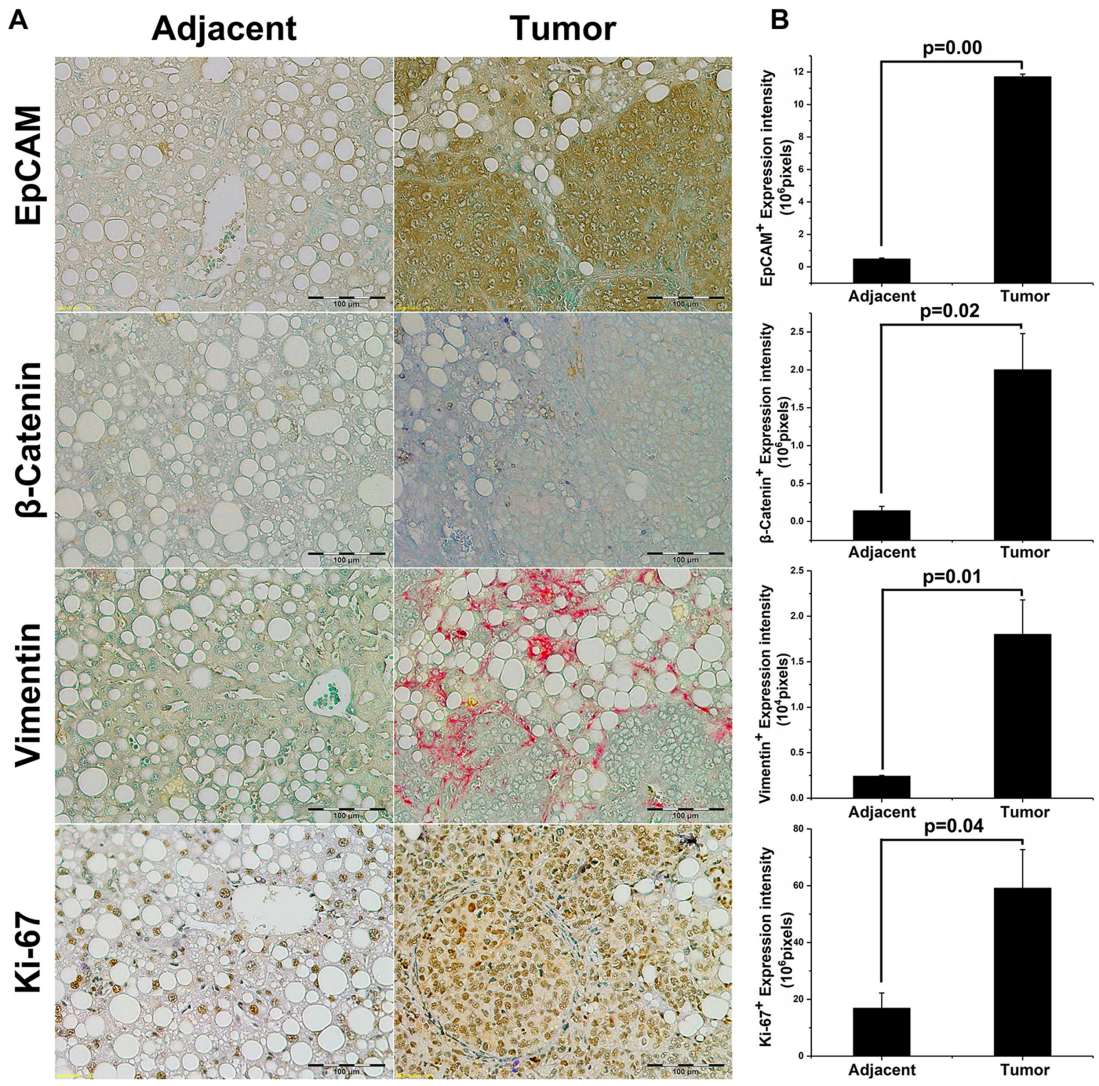
Tumor initiation in EpCAM+ NASH animals exhibits aggressive HCC tumorigenesis. IHC-P staining was performed in paraffin-fixed tumor and liver tissues. Four specific markers correlated with poor prognosis and tumorigenesis were evaluated: EpCAM, β-Catenin, Vimentin, and Ki-67. (**A**) Representative IHC-P staining slides for each experimental group and marker; (**B**) Box plot showing quantified expression comparison of adjacent and tumor tissue.

We analyzed orthotopic tumors in EpCAM+ group NASH animals by IHC-P and compared them with adjacent non-tumor liver. Figure 3A shows representative IHC-P images, while Figure 3B shows the quantification of expression in IHC slides. EpCAM expression was significantly higher in tumor tissue compared with adjacent tissue [Mean total positive intensity (10^6 pixels); Tumor = 11.7 ± 0.17, Adjacent = 0.48 ± 0.7, *p* = 0.0000000303]. EpCAM is a downstream target of canonical Wnt/β-catenin pathway, and β-catenin also was reported to correlate with poor prognosis in HCC patients [21, 22]. Therefore, we further analyzed β-catenin expression. Tumor tissues showed significant higher levels of β-catenin expression compared with adjacent nontumor tissue [Mean total positive intensity (10^6 pixels); Tumor = 2.0 ± 0.48, Adjacent = 0.14 ± 0.06, *p* = 0.02]. Interestingly, the IHC suggested that β-catenin was no longer restricted to membrane-bound but also found in both cytoplasm and nuclei in tumor tissues and the EpCAM+ CSCs -were growing in whole liver beyond site of. Therefore, we analyzed Vimentin – a well-characterized EMT marker [23]. The results showed that Vimentin expression was significantly higher in tumor tissue compared with adjacent nontumor tissue [Mean total positive intensity (10^4 pixels); Tumor = 1.8 ± 0.38, Adjacent = 0.24 ± 0.1, *p* = 0.01]. Increased Ki-67 expression by IHC analysis was reported as HCC progression and poor prognosis in patients [22, 24]. These tumors showed significant higher Ki-67 expression compared with adjacent nontumor tissue [Mean total positive intensity (10^6 pixels); Tumor = 59.1 ± 13.6, Adj = 16.8 ± 5.4, *p* = 0.035]. Taken together, the EpCAM+ CSC-initiated tumor growth in the NASH microenvironment represent an aggressive form of HCC tumorigenesis.

### Lineage tracking showed that tumorigenesis primarily occurs from EpCAM+ CSCs

**Figure 4:**
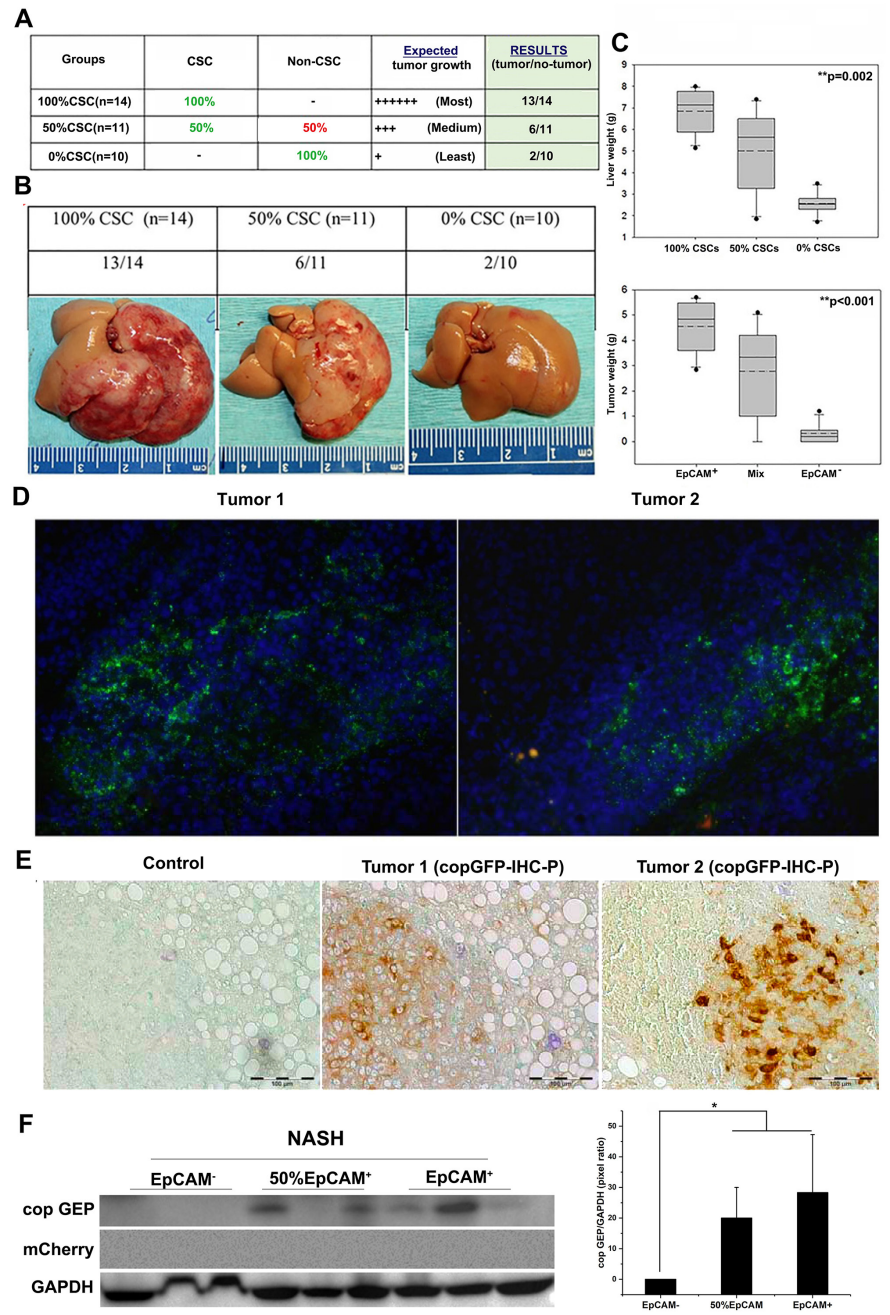
Lineage tracking study showed that tumor initiation in NASH liver microenvironment is dose-dependent of EpCAM+ CSCs. NASH group mice were orthotopically injected with 2 million Hepa1-6 cells. (**A**) Table showing experimental scheme of 3 groups, expected and observed results. (**B**) Representative gross liver pictures with tumor burden for each of the 3 groups of NASH mice. (**C**) Upper box plot showing whole liver weight, and lower box plot showing tumor weight, for each experimental group. EpCAM+ group (100% EpCAM+ CSCs) showed significant higher liver weight (6.86 ± 0.42 g, *p* = 0.002) and tumor weight (4.56 ± 0.42 g, *p* < 0.001), followed by mix group with 50% EpCAM+ CSCs (Liver: 5.00 ± 0.73 g, and tumor: 2.77 ± 0.69 g), and lowest in EpCAM- group with 0% CSCs, (Liver: 3.13 ± 0.59 g, and tumor: 0.8 ± 0.50 g). Solid lines in box plot represent median and dotted lines represent arithmetic mean of each groups. Significance was calculated by one-way ANOVA, and *p* value is shown on upper right corner of each box plots. (**D**) IHC-Fz staining and analysis of two different tumors from the mixed group (50:50, EpCAM+: EpCAM-) in orthotopic NASH mouse model. Majority of tumors showed with green fluorescence (copGFP tagged EpCAM+ CSCs), while only very minute fraction of tumor showed red fluorescence in tumor # 2 (mCherry tagged EpCAM- non-CSCs). (**E**) IHC-P staining confirmed copGFP expression using anti-copGFP antibody staining with secondary DAB color development with HRP-polymer. (**F**) Western Blot analysis confirmed copGFP expression from tumors of the mixed group and EpCAM+ group, while no mCherry expression was detected in mixed group tumors. ^*^ *p* < 0.05.

We designed a lineage-tracking study with well-defined objectives, i. e., (1) identify the source of tumor initiation in immunocompetent mice, and (2) systematically evaluate potential contributions of EpCAM+ and EpCAM- Hepa1-6 cells as well as the host liver cells to tumorigenesis in NASH liver. We used 3 group of cells, defining as 1) 100% CSC (100% EpCAM+ Hepa1-6 cells); 50% CSC (50/50 ratio of mixed EpCAM+/EpCAM- Hepa1-6 cells; and 0% CSC (100% EpCAM- Hepa1-6 cells) for orthotopic inoculation with total 2 × 10^6^ cells. (Figure 4A). In group 1 and 3, both of EpCAM+/EpCAM- Hepa1-6 cells are stably expressing copGFP and total while in group 2, 1 × 10^6^ EpCAM+ cells stably expressing copGFP and 1 × 10^6^ EpCAM- Hepa1-6 cells stably expressing mCherry were mixed and injected into left liver lobes of animals using the orthotopic injection procedure previously described.

Tumor incidence and tumor burden were found to be dependent on the injected numbers of EpCAM+ Hepa1-6 cells (Figure 4B). The liver weight and tumor weight were further determined. As shown in Figure 4C, the EpCAM+ group (100% EpCAM+ CSCs) showed significantly higher liver weight (6.86 ± 0.42 g, *p* = 0.002), followed by the mixed group with 50% EpCAM+ CSCs (5.00 ± 0.73 g), and lowest in the EpCAM- group with 0% CSCs, (3.13 ± 0.59 g). Similarly, tumor weights were significantly higher in the EpCAM+ group (4.56 ± 0.42 g, *p* < 0.001), followed by the mixed group with 50% EpCAM+ CSCs (2.77 ± 0.69 g), and least in the EpCAM- group with 0% CSCs, (0.8 ± 0.50 g). The mixed group’s tumors were of the highest interest for us, because these tumors were initiated from a 50/50 mixture of copGFP-expressing EpCAM+ CSCs and mCherry-expressing EpCAM- non-CSCs (Hepa1-6 cells) to track the tumor lineage *in vivo*. Two tumors from different animals were analyzed by IHC-Fz analysis (Figure 4). Tumor # 2 showed a faint expression of mCherry, but not significant, thus suggesting EpCAM+ cells (copGFP) dominated tumor growth within the tumor microenvironment over EpCAM- non-CSCs (mCherry). We confirmed the copGFP expression by IHC-P analysis (Figure 4E). We further confirmed the expressions of copGFP and mCherry by western blot analysis (Figure 4F). We only observed copGFP expression in the mixed group and EpCAM+ groups, but not in the EpCAM- group. Similarly, no expression was detected for mCherry in the mixed group in Western blot analysis. Taken together, we demonstrated that EpCAM+ Hepa1-6 CSCs possess predominant tumor-initiating properties in the NASH microenvironment of immunocompetent mice.

### Degree of EpCAM expression of Hep3B and HepG2 cells influences tumorigenesis in orthotopic Nu/J mice livers

Although the HCC cell line Hep3B has been studied in orthotopic liver microenvironments which show characteristics of human HCC with detectable plasma levels of AFP in BALB/C nude mice [25], no previous study tested if EpCAM expression could affect the tumor-initiating ability of Hep3B cells in an orthotopic mice model. Therefore, we systematically studied the ability of EpCAM-Low and EpCAM-High CSCs in liver microenvironments using an orthotopic mouse model.

**Figure 5:**
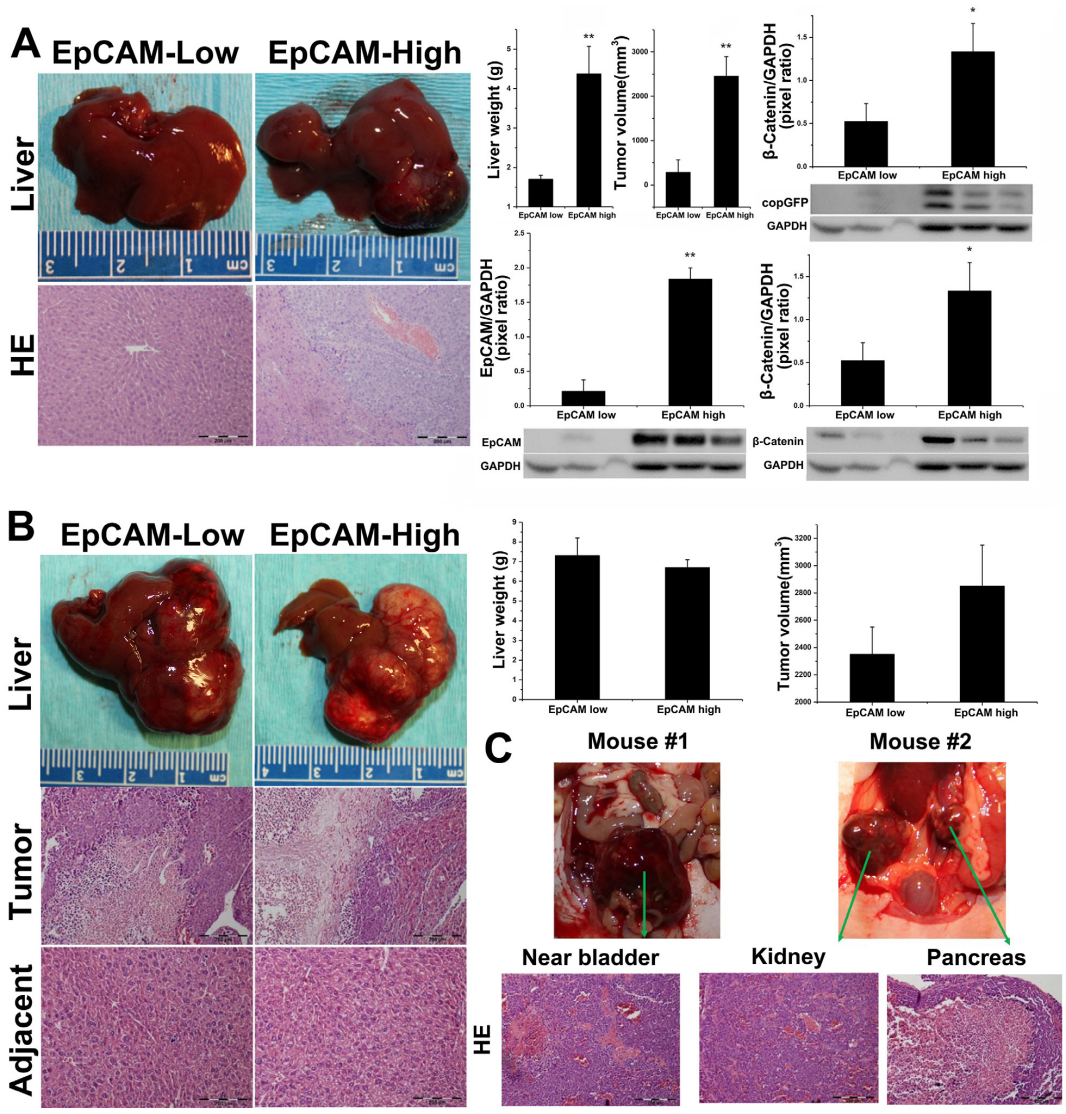
Hep3B and HepG2 evaluation in orthotopic Athymic Nu/J mouse models. EpCAM-High Hep3B subset possess tumorigenic potential in orthotopic Nu/J mouse model. 2 million sorted EpCAM-low or EpCAM-high Hep3B cells with constitutive copGFP expression were injected into left liver lobe of athymic Nu/J nude mice with survival surgery, and tumor growth was monitored for 70 Days (*n* = 4 mice/group). (**A**) left: Gross liver and H&E stain of EpCAM-low and EpCAM-High animals. All 4/4 animals in EpCAM-high group confirmed tumor by H&E staining, while only 1/4 animals in EpCAM-low group showed minor tumor in injection lob. Right: EpCAM-high group showed significant higher liver weight and tumor volume compared with EpCAM-low groups. Error bar = SEM, ^*^ *p* < 0.01, ^**^ *p* < 0.001; Western blot analysis showed that EpCAM-high group animals/tumors show significant higher β-catenin and concomitant EpCAM expression, with copGFP co-expression; Densitometric analysis of β-catenin (^*^ *p* < 0.05) and EpCAM (^**^ *p* < 0.001). GAPDH is used as an internal control to normalize expression. For HepG2, 2 million sorted EpCAM-low or EpCAM-high HepG2 cells with constitutive copGFP expression were injected into left liver lobe of athymic Nu/J nude mice with survival surgery, and tumor growth was monitored for 70 Days. (**B**) Left: Gross anatomy of liver lobes and H&E staining of EpCAM-Low and EpCAM-High animals. All 4/4 animals in EpCAM-Low and EpCAM-High group confirmed tumor by H&E staining. Right: There was no significant difference observed in liver weight and tumor volume between both groups. Total *n* = 4 mice/group, *p* = NS. (**C**) EpCAM-High HepG2 cells metastasize in orthotopic athymic Nu/J mouse model: Two of the four animals had confirmed metastases of HepG2. In the first animal, a metastasis was observed near bladder, while the second animal showed metastatic disease in kidney and pancreas. Lower panel shows H&E staining of metastatic tissues, confirming tumors.

A total of 2 million EpCAM-Low or EpCAM-High Hep3B cells (stably expressing copGFP) were sorted by FACS and injected into left liver lobes of 10-week-old athymic Nu/J nude mice as described above (*n* = 4 mice/group, two groups, EpCAM-Low and EpCAM-High). Tumor growth was monitored using non-invasive ultrasound imaging, and the tumor nodule was detected on Day 35 in the EpCAM-High group (data not shown). Mice were followed for 70 days and euthanized (all mice were survived for 70 days in both groups). Macroscopically, all 4 mice in EpCAM-High group had tumor growth confirmed by ultrasound, while only 1 out of 4 mice in EpCAM-Low group were confirmed with tumor growth (significantly lower in tumor size and volume compared with EpCAM-High group tumors). As shown in Figure 5A, gross liver anatomy and H&E staining confirmed HCC tumor growth in the EpCAM-High group of animals. The EpCAM-High group of animals showed significantly higher tumor volume (2451.25 ± 443.34 mm^3^, *p* = 0.013, *n* = 4 mice/group) and significantly higher liver weight (4.375 ± 0.7 g, *p* = 0.023, *n* = 4 mice/group) compared to the EpCAM-Low group (tumor volume: 282.22 ± 282 mm^3^, liver weight:1.7 ± 0.1 g). We further analyzed expression of β-catenin, EpCAM, and copGFP by Western blot. As shown in Figure 5A, EpCAM-High group tumors confirmed copGFP expression suggesting tumors were initiated from the injected EpCAM cells.

HepG2, a unique HCC cell line, correlated with high Wnt/β-catenin HCC subtype in patients due to somatic mutation leading to a truncated β-catenin, which is constitutively active [26, 27]. As EpCAM expression is regulated by Wnt/β-catenin pathway, the fate of HepG2 cell line in orthotopic liver microenvironments could be very distinct compared with Hepa1-6 or Hep3B cell lines. The experimental strategy described for the Hep3B experiments above was followed for the HepG2 orthotopic experiments. Detectable tumors were found on Day 30 by ultrasound in both EpCAM-High and EpCAM-Low group animals. Mice were followed for 70 days for tumor growth and animals were euthanized. As shown in Figure 5B, all mice in both EpCAM-High and EpCAM-Low groups confirmed presence of tumors by gross liver and H&E histology analysis. No significant differences were observed in liver weight and tumor volume between these two groups. Interestingly, metastatic HCC occurred in 2/4 EpCAM-High animals (Figure 5C), and all the tumors were confirmed by H&E histology analysis. In one mouse, metastatic tumor was observed near to bladder (overlapping with bladder tissue and connecting tissues), and in the second mouse, we found two distinct metastatic tumors (right kidney and pancreas). Taken together, we have shown that the tumor-initiation capabilities of HepG2 cells were not significantly affected by EpCAM expression *in vivo* in an orthotopic liver microenvironment. However, EpCAM-High HepG2 cells acquired metastatic abilities, suggesting that EpCAM could play a unique role contributing to tumorigenesis despite constitutively active Wnt/β-catenin signaling due to predominant mutant β-catenin in HepG2 cells.

## DISCUSSION

In this study, extensive and comprehensive *in vivo* studies were performed. The results of orthotopic models confirmed that higher tumorigenic capabilities were characteristic of the EpCAM-expressing CSCs in liver microenvironment. We established mouse models representing 3 distinct diet-induced liver microenvironments: healthy (control diet), bland steatosis (HFD), and inflammatory steatohepatitis (NASH diet). Then, we systematically studied tumor-initiation abilities of EpCAM+ CSCs and EpCAM- Hepa1-6 cells in livers of animals by orthotopic inoculation. Control and HFD group of animals failed to initiate tumor, suggesting that healthy liver and bland-steatosis liver did not render an appropriate microenvironment for EpCAM+ CSCs survival. However, in the NASH microenvironment, EpCAM+ CSCs showed aggressive tumor growth while EpCAM- Hepa1-6 cells demonstrated incidental tumor initiation (2/10 mice with tumors, much smaller in size and volume compared to EpCAM+ tumor). IHC-P analysis of tumors from EpCAM+ NASH animals further confirmed significant higher EpCAM, β-catenin, Ki-67, and Vimentin, thus suggesting that HCC tumor growth in NASH animals by EpCAM-expressing Hepa1-6 was highly tumorigenic. We also performed lineage-tracking studies to identify the source of tumor initiation in the NASH microenvironment. Our findings in lineage-tracking studies showed that HCC initiation in C57L/J NASH mice was predominantly from EpCAM+ CSCs and not from EpCAM- Hepa1-6 cells.

Next, we evaluated two human EpCAM-expressing cell lines (Hep3B and HepG2). In mice with Hep3B inoculation, the EpCAM-High group confirmed aggressive tumor incidence (*n* = 4/4), while the EpCAM-Low group showed only *n* = 1/4 mouse with successful but significant small tumor size. The increased levels of EpCAM and β-catenin in EpCAM-High group suggest that EpCAM-High Hep3B cells preserved their inherent expression and properties *in vivo* within liver microenvironments and were responsible for tumor initiation. Absence of tumor and copGFP/EpCAM expression in the EpCAM-Low group suggests that Hep3B cells with lower EpCAM expression do not carry tumor-initiation capability *in vivo*. For the first time, we demonstrate that, without genetic alteration, high levels of EpCAM expression on Hep3B cells possess unique tumor-initiating capability in the liver microenvironment, and thus possibly contribute distinctly to tumorigenesis. Unlike Hep3B, the HepG2 experiment failed to exhibit differences in tumor volume or liver weight between EpCAM-High and EpCAM-Low groups. Orthotopic tumors were observed with high necrotic regions and soft fluid-filled regions, representing human HCC qualities. However, 2/4 EpCAM-High animals had confirmed metastasis (kidney and pancreas). Our results demonstrate that EpCAM expression of HepG2 cells did not affect its tumorigenic abilities, but potentially facilitates metastasis in the liver microenvironment. These results were interesting to us because HepG2 has not been reported as a metastatic cell line.

Clinical trials targeting the CSC activation pathway face challenges due to an incomplete understanding of stemness plasticity in cancer cells [28]. Clinical trials rely on data obtained from pre-clinical rodent models, and a majority of current CSC knowledge was developed from athymic or SCID mice with hind-limb tumor xenografts [29]. There remains an unmet need to test the CSC properties in orthotopic rodent models with clinical relevance.

The estimated annual HCC incidence in NASH is about 0.3% [30]. Of interest is that HCC has been increasingly recognized in NASH patients without cirrhosis [31, 32]. In our previous animal studies, we found NASH associated HCC initiation of HCC [33, 34]. In this study, we tested the CSCs derived from HCC cell lines in orthotopic liver mouse models. Hepa1-6 is a murine HCC cell line closely representing poorly differentiated HCC growth in C57BL/6J and C57L/J mouse models. It has already been established that fatty liver progression exacerbates HCC carcinogenic events within liver microenvironments [35]. We demonstrate that the NASH liver is favorable to EpCAM+ CSC-mediated tumorigenesis in immunocompetent microenvironments, but not the normal liver or the nonprogressive bland steatosis. Importantly, our findings suggest that EpCAM+ CSCs may successfully evade host immune surveillance in the NASH liver microenvironment. Therefore, the NASH mouse model could serve as a vital tool to study immune-checkpoint therapies and immune surveillance in tumor immunology.

The Hep3B cell line is p53-null with mesenchymal phenotype, slow growing, and demonstrates well-differentiated HCC tumors in mouse models [25, 36]. Orthotopic evaluation of EpCAM-High and EpCAM-Low subsets of Hep3B demonstrated that the levels of EpCAM expression, rather than merely its presence or absence, positively correlates with severity of tumorigenesis. Hep3B/Nu-J experiments provide glimpses into the nature of EpCAM-expressing CSCs that hierarchal EpCAM expression bears different tumor initiating capability. Unlike previous studies, we did not observe any metastasis in Hep3B-inoculated animals.

The HepG2 cell line expresses a constitutively active form of β-catenin (truncated β-catenin, ~75 KD); however, it is considered to be a nonneoplastic hepatoma cell line [26, 27] because few studies have been successful in tumor initiation in animal models [10, 37]. Therefore, we tested HepG2 EpCAM-High and EpCAM-Low subsets in orthotopic animal models and found no significant differences in tumorigenesis potential. Considering constitutively active β-catenin status in HepG2 as explained above, distinct outcomes of HepG2/Nu-J experiments are not surprising. However, this study is first to report that the EpCAM-High HepG2 subset possesses metastatic ability. Interestingly, HepG2/Nu-J experiments demonstrated that despite a constitutively active β-catenin, higher degrees of EpCAM expression can steer these cells towards metastasis; further study is needed to unveil the potential mechanism.

In conclusion, we demonstrated that the NASH liver microenvironment promotes EpCAM+ CSC-mediated HCC tumor initiation in an immunocompetent mouse model, thus recapitulating clinical conditions. An athymic mouse model with orthotopic inoculation of Hep3B and HepG2 cells provides evidence on how different levels of EpCAM expression contribute to HCC tumorigenesis in liver microenvironments. The methodology we established for using orthotopic HCC models is clinically relevant and may provide a better pre-clinical platform to evaluate HCC tumorigenesis as well as potential anti-cancer drug candidates. Future studies are encouraged in this direction.

## MATERIALS AND METHODS

### Human HCC specimens and TCGA analysis

This study was approved by the Institutional Review Board (IRB) for human study at the University of Louisville. The study samples for IHC were retrospectively collected from 24 patients who had undergone liver resection for hepatocellular carcinoma (HCC) between 2002 and 2013. In total 24 subjects were identified, 13 male (54.2%) and 11 female (45.8%), with a median age of 67, ranging from 41 to 84 years old. Eight patients had clinical diagnoses of HCC and fatty liver and the liver resection samples were selected for Western blot analysis. The control samples consisted of the same 24 patients’ normal adjacent tissue that were within the resected liver specimen. The Cancer Genome Atlas (TCGA database) analysis was carried out at https://portal.gdc.cancer.gov.

### Cell lines

Hepatoma cell lines Hepa1-6 (mouse), Hep3B and HepG2 (human) were obtained from an American-type culture collection (Manassas, USA). Hepa1-6 cells were grown in DMEM with 4.5% Glucose, 10% FBS and 1× antibiotic-antimycotic. HepG2 and Hep3B cells were grown in MEM with 10% FBS, 1× nonessential amino acids, 1× sodium pyruvate, and 1× antibiotic-antimycotic. Spheroid assay was performed in all 3 cell lines to induce SCSs formation as previous reported [20]. All cell lines were validated every six months or obtained a new ATCC stock every 6 months.

### Lentivirus transduction and generation of stable HCC cell lines

Fourth-generation lentivirus packing systems (Lenti-X, Takara-Clontech) were used to generate stable copGFP-expressing and mCherry-expressing cell lines. Briefly, puromycin-resistant – copGFP (pLenti-CMV-GFP-Puro) and blasticidin-resistant mCherry (pLV-Bsd-CMV-mCherry) vectors were obtained and amplified in stable competent EColi (New England Biolabs) by employing standard molecular biology protocol with 100 µg/mL Ampicillin selection, purified for lentivirus packing using commercial plasmid prep kit (Takara-Clontech). For generating lentivirus, Lenti-X reagent was mixed with 6 µg of lentivirus plasmid and transfected in 293T cell line (Takara-Clontech) for packing. Harvested lentivirus was used to transduce target cell lines using polybrene by following established protocol. Hepa1-6, HepG2, and Hep3B cell lines were transduced with copGFP lentivirus, and cell lines were selected with 3 µg/mL puromycin (Sigma-Aldrich) and expanded after confirmation by fluorescence microscope for copGFP expression (Supplementary Figure 4). Hepa1-6 cell line was transduced with mCherry lentivirus, and selected with 10 µg/mL blasticidin (Sigma-Aldrich).

### Fluorescence assisted cell sorting (FACS)

Hepa1-6, HepG2, and Hep3B cells were cultured to 80% confluency by employing ATCC-recommended cultural methods in T75 tissue culture treated flasks (TPP, Switzerland). On the day of cell sorting, cells were trypsinized (Corning) and viable cells were determined using trypan blue and haemocytometer. Cells were stained using APC conjugated Anti-EpCAM antibody (Miltenyi Biotech, Germany) per manufacturer instructions in the dark. Before cell sorting, each cell preparation was filtered through a 40-µm sieve (VWR) and kept on ice. Cell sorting was performed using BD MoFlo cell sorter. Sorted cells were collected in complete media with 10% FBS, washed, and cultured back in fresh complete media with 10% FBS at 37°C incubator with 5% CO_2_. To remove bound anti-EpCAM antibodies and bring cells back in log phase, sorted cells were grown for 3–4 days after sorting, and used for orthotopic injection.

### Animal experiments

All experimental procedures were approved by the Institutional Animal Care and Use Committee (IACUC) at the University of Louisville. The mice were housed in the UofL Research Resources Center at 22°C with 12-hour light/dark cycles with free access to food and water.

### Establishing immunocompetent orthotopic animal models with diet-induced liver microenvironment

Sexually matured 8-week-old C57L/J mice (Stock No: 000668, Jackson Laboratory, USA) were inbred to develop consistent and accurate diet-induced mouse models necessary for this study as previously described [38]. Briefly, after 1 month from the birth, mice were randomly assigned to one of three groups (*n* = 8 mice/group). Then each group was fed with its respective diet: normal control diet (10% fat, D12450B, Research Diets, Inc., New Brunswick, NJ); high-fat diet (60% fat, D12492, Research Diets, Inc., New Brunswick, NJ); choline deficient, L-amino acid defined high-fat diet with 0.1% methionine (CDAHFD, 60% fat, A06071302, Research Diets, Inc., New Brunswick, NJ) for the next 10 weeks. For orthotopic inoculation, 2 × 10^6^ sorted EpCAM+ or EpCAM- Hepa1-6 cells were injected into left liver lobe of the mouse. Evaluating EpCAM+ CSCs derived from Hepa1-6 in the above mentioned 3 different animal models exhibiting 3 distinct liver pathologies (control diet: healthy, HFD: bland steatosis, and CDAHFD: steatohepatitis, respectively) allowed us to systematically investigate the tumorigenic capability of hepatocellular carcinoma CSCs in different immunocompetent liver microenvironments. Post injection, mice were monitored by high-frequency ultrasound to follow the tumor growth and all animals were euthanized on day 18. Animal weight, liver weight, tumor weight, and tumor size were recorded for each animal. NASH group experiments were entirely repeated 2 more times independently to confirm findings (at least *n* = 3 animals in each experimental group/attempt).

### Establishing athymic orthotopic mouse models (for HepG2 and Hep3B studies)

10 week-old male Nu/J mice (Stock No: 002019, Jackson laboratory, USA) were used for both HepG2 and Hep3B *in vivo* experiments. For orthotopic inoculation, 2 × 10^6^ sorted EpCAM-Low or EpCAM-High cells (HepG2 or Hep3B) were injected into left liver lobe of an animal. Post injection, mice were monitored by high frequency ultrasound for tumor growth, and all animals were euthanized on day 70. Animal weight, liver weight, tumor weight, and tumor size were recorded for each animal.

### Non-invasive orthotopic tumor monitoring by high frequency ultrasound

To non-invasively assess orthotopic liver tumor growth *in vivo*, we utilized a high frequency ultrasound method using VisualSonics Vevo2100 system with MS-400 probe (30-MHz center frequency) as previously demonstrated [38]. B-mode imaging data were acquired at defined time-points. For the immunocompetent mouse model using Hepa1-6 and C57L/J mice, we recorded baseline, D5, D9, and D13. For athymic mouse models using HepG2 and Hep3B with Nu/J mouse, we recorded baseline, D5, D15, D25, D40, D70.

### Histology procedure and analysis

After animals were euthanized, liver tissues were isolated and weighted. A piece of tissue was taken from a liver lobe and fixed in 10% buffered formalin for 24 hours and transferred to 80% ethanol. The formalin-fixed liver tissue was processed (stepwise dehydrated) and embedded in paraffin by following standard procedure. Serial 5-µm sections were mounted onto glass slides. These slides were used for hematoxylin and eosin (H&E) staining and immunohistochemistry (IHC) staining for each animal.

### Hematoxylin and eosin (H&E) staining and analysis

HE staining was blinded and analyzed by two pathologists for experimental groups and assigned a total NAFLD histopathology score (0–8) based on inflammation (0–3), steatosis (0–3), and hepatocyte ballooning (0–2) [39, 40].

### IHC staining and analysis

IHC staining was performed by using DAKO overnight incubation of primary antibody in darkness at 4°C (1:100 dilution). On the second day, after washing primary antibody, staining was developed by performing DAB conjugated polymers. All antibody source, catalog information, and dilutions are provided in Supplementary Tables 1 and 2. Analysis was performed by Aperio ImageScope software (Leica Biosystems) by employing a pixel density algorithm. Total positive intensity (pixels) was represented by box plot. Statistical comparison was performed using two-tail Student’s *t*-test assuming equal variance.

### Statistical analysis

Data are presented as mean ± S. D or mean ± SEM (*n* ≥ 3 per group). Comparison statistics were performed by two-tail student’s *t*-test with equal variance or one-way analysis of variance (ANOVA). All the statistical analysis was performed (including chart, plots etc.) using either SigmaPlot statistical software or Microsoft Excel 2013 (Redmond, USA). Results with *p* ≤ 0.05 were considered statistically significant.

## SUPPLEMENTARY MATERIALS



## Abbreviations

ALDH1: aldehyde dehydrogenase 1
AFP: alpha-fetoprotein
CSC: cancer stem cells
CTNNB1: β-catenin gene
EpCAM: epithelial cell adhesion molecule
GEPIA: Gene Expression Profiling Interactive Analysis
HCC: hepatocellular carcinoma
NAFLD: Non-alcoholic fatty liver disease
NASH: non-alcoholic steatohepatitis

## References

[1] Altekruse SF , McGlynn KA , Reichman ME . Hepatocellular carcinoma incidence, mortality, and survival trends in the United States from 1975 to 2005. J Clin Oncol. 2009; 27:1485–1491. 10.1200/JCO.2008.20.7753. 19224838 PMC2668555

[2] Parkin DM , Bray F , Ferlay J , Pisani P . Global cancer statistics, 2002. CA Cancer J Clin. 2005; 55:74–108. 10.3322/canjclin.55.2.74. 15761078

[3] El-Serag HB . Hepatocellular Carcinoma. N Engl J Med. 2011; 365:1118–1127. 10.1056/NEJMra1001683. 21992124

[4] NCI. Surveillance, Epidemiology, and End Results (SEER) Program (https://seer.cancer.gov: Natinal Cancer Institute). 2016.

[5] Chiba T , Kamiya A , Yokosuka O , Iwama A . Cancer stem cells in hepatocellular carcinoma: Recent progress and perspective. Cancer Lett. 2009; 286:145–153. 10.1016/j.canlet.2009.04.027. 19464789

[6] Ma S , Chan KW , Hu L , Lee TK , Wo JY , Ng IO , Zheng BJ , Guan XY . Identification and characterization of tumorigenic liver cancer stem/progenitor cells. Gastroenterology. 2007; 132:2542–2556. 10.1053/j.gastro.2007.04.025. 17570225

[7] Yoon SK . The biology of cancer stem cells and its clinical implication in hepatocellular carcinoma. Gut Liver. 2012; 6:29–40. 10.5009/gnl.2012.6.1.29. 22375168 PMC3286736

[8] Liu LL , Fu D , Ma Y , Shen XZ . The power and the promise of liver cancer stem cell markers. Stem Cells Dev. 2011; 20:2023–2030. 10.1089/scd.2011.0012. 21651381

[9] Ma S . Biology and clinical implications of CD133(+) liver cancer stem cells. Exp Cell Res. 2013; 319:126–132. 10.1016/j.yexcr.2012.09.007. 22999864

[10] Zhao X , Parpart S , Takai A , Roessler S , Budhu A , Yu Z , Blank M , Zhang YE , Jia HL , Ye QH , Qin LX , Tang ZY , Thorgeirsson SS , et al. Integrative genomics identifies YY1AP1 as an oncogenic driver in EpCAM(+) AFP(+) hepatocellular carcinoma. Oncogene. 2015; 34:5095–5104. 10.1038/onc.2014.438. 25597408 PMC4506915

[11] Terris B , Cavard C , Perret C . EpCAM, a new marker for cancer stem cells in hepatocellular carcinoma. J Hepatol. 2010; 52:280–281. 10.1016/j.jhep.2009.10.026. 20006402

[12] Yamashita T , Forgues M , Wang W , Kim JW , Ye Q , Jia H , Budhu A , Zanetti KA , Chen Y , Qin LX , Tang ZY , Wang XW . EpCAM and alpha-fetoprotein expression defines novel prognostic subtypes of hepatocellular carcinoma. Cancer Res. 2008; 68:1451–1461. 10.1158/0008-5472.CAN-07-6013. 18316609

[13] Yamashita T , Honda M , Nakamoto Y , Baba M , Nio K , Hara Y , Zeng SS , Hayashi T , Kondo M , Takatori H , Yamashita T , Mizukoshi E , Ikeda H , et al. Discrete nature of EpCAM+ and CD90+ cancer stem cells in human hepatocellular carcinoma. Hepatology. 2013; 57:1484–1497. 10.1002/hep.26168. 23174907 PMC7180389

[14] Yamashita T , Ji J , Budhu A , Forgues M , Yang W , Wang HY , Jia H , Ye Q , Qin LX , Wauthier E , Reid LM , Minato H , Honda M , et al. EpCAM-positive hepatocellular carcinoma cells are tumor-initiating cells with stem/progenitor cell features. Gastroenterology. 2009; 136:1012–1024. 10.1053/j.gastro.2008.12.004. 19150350 PMC2828822

[15] Yamashita T , Wang XW . Cancer stem cells in the development of liver cancer. J Clin Invest. 2013; 123:1911–1918. 10.1172/JCI66024. 23635789 PMC3635728

[16] Goldberg D , Ditah IC , Saeian K , Lalehzari M , Aronsohn A , Gorospe EC , Charlton M . Changes in the Prevalence of Hepatitis C Virus Infection, Nonalcoholic Steatohepatitis, and Alcoholic Liver Disease Among Patients With Cirrhosis or Liver Failure on the Waitlist for Liver Transplantation. Gastroenterology. 2017; 152:1090–1099.e1. 10.1053/j.gastro.2017.01.003. 28088461 PMC5367965

[17] Lazo M , Hernaez R , Eberhardt MS , Bonekamp S , Kamel I , Guallar E , Koteish A , Brancati FL , Clark JM . Prevalence of nonalcoholic fatty liver disease in the United States: the Third National Health and Nutrition Examination Survey, 1988–1994. Am J Epidemiol. 2013; 178:38–45. 10.1093/aje/kws448. 23703888 PMC3698993

[18] McCullough AJ . Epidemiology of the metabolic syndrome in the USA. J Dig Dis. 2010; 12:333–340. 10.1111/j.1751-2980.2010.00469.x. 21091931

[19] Michelotti GA , Machado MV , Diehl AM . NAFLD, NASH and liver cancer. Nat Rev Gastroenterol Hepatol. 2013; 10:656–665. 10.1038/nrgastro.2013.183. 24080776

[20] Pandit H , Li Y , Li X , Zhang W , Li S , Martin RCG . Enrichment of cancer stem cells via β-catenin contributing to the tumorigenesis of hepatocellular carcinoma. BMC Cancer. 2018; 18:783. 10.1186/s12885-018-4683-0. 30075764 PMC6091111

[21] Inagawa S , Itabashi M , Adachi S , Kawamoto T , Hori M , Shimazaki J , Yoshimi F , Fukao K . Expression and prognostic roles of beta-catenin in hepatocellular carcinoma: correlation with tumor progression and postoperative survival. Clin Cancer Res. 2002; 8:450–456. 11839663

[22] Srivastava S , Thakkar B , Yeoh KG , Ho KY , Teh M , Soong R , Salto-Tellez M . Expression of proteins associated with hypoxia and Wnt pathway activation is of prognostic significance in hepatocellular carcinoma. Virchows Arch. 2015; 466:541–8. 10.1007/s00428-015-1745-4. 25742908

[23] Hu L , Lau SH , Tzang CH , Wen JM , Wang W , Xie D , Huang M , Wang Y , Wu MC , Huang JF , Zeng WF , Sham JS , Yang M , et al. Association of Vimentin overexpression and hepatocellular carcinoma metastasis. Oncogene. 2004; 23:298–302. 10.1038/sj.onc.1206483. 14647434

[24] Stroescu C , Dragnea A , Ivanov B , Pechianu C , Herlea V , Sgarbura O , Popescu A , Popescu I . Expression of p53, Bcl-2, VEGF, Ki67 and PCNA and prognostic significance in hepatocellular carcinoma. J Gastrointestin Liver Dis. 2008; 17:411–417. 19104702

[25] Yao X , Hu JF , Daniels M , Yien H , Lu H , Sharan H , Zhou X , Zeng Z , Li T , Yang Y , Hoffman AR . A novel orthotopic tumor model to study growth factors and oncogenes in hepatocarcinogenesis. Clin Cancer Res. 2003; 9:2719–2726. 12855652

[26] Carruba G , Cervello M , Miceli MD , Farruggio R , Notarbartolo M , Virruso L , Giannitrapani L , Gambino R , Montalto G , Castagnetta L . Truncated form of beta-catenin and reduced expression of wild-type catenins feature HepG2 human liver cancer cells. Ann N Y Acad Sci. 1999; 886:212–216. 10.1111/j.1749-6632.1999.tb09419.x. 10667222

[27] de La Coste A , Romagnolo B , Billuart P , Renard CA , Buendia MA , Soubrane O , Fabre M , Chelly J , Beldjord C , Kahn A , Perret C . Somatic mutations of the beta-catenin gene are frequent in mouse and human hepatocellular carcinomas. Proc Natl Acad Sci U S A. 1998; 95:8847–8851. 10.1073/pnas.95.15.8847. 9671767 PMC21165

[28] Ramos EK , Hoffmann AD , Gerson SL , Liu H . New Opportunities and Challenges to Defeat Cancer Stem Cells. Trends Cancer. 2017; 3:780–796. 10.1016/j.trecan.2017.08.007. 29120754 PMC5958547

[29] Bruttel VS , Wischhusen J . Cancer Stem Cell Immunology: Key to Understanding Tumorigenesis and Tumor Immune Escape? Front Immunol. 2014; 5:360. 10.3389/fimmu.2014.00360. 25120546 PMC4114188

[30] Zoller H , Tilg H . Nonalcoholic fatty liver disease and hepatocellular carcinoma. Metabolism. 2016; 65:1151–1160. 10.1016/j.metabol.2016.01.010. 26907206

[31] Guzman G , Brunt EM , Petrovic LM , Chejfec G , Layden TJ , Cotler SJ . Does nonalcoholic fatty liver disease predispose patients to hepatocellular carcinoma in the absence of cirrhosis? Arch Pathol Lab Med. 2008; 132:1761–1766. 18976012 10.5858/132.11.1761

[32] Leung C , Yeoh SW , Patrick D , Ket S , Marion K , Gow P , Angus PW . Characteristics of hepatocellular carcinoma in cirrhotic and non-cirrhotic non-alcoholic fatty liver disease. World J Gastroenterol. 2015; 21:1189–96. 10.3748/wjg.v21.i4.1189. 25632192 PMC4306163

[33] Cui G , Martin RC , Jin H , Liu X , Pandit H , Zhao H , Cai L , Zhang P , Li W , Li Y . Up-regulation of FGF15/19 signaling promotes hepatocellular carcinoma in the background of fatty liver. J Exp Clin Cancer Res. 2018; 37:136. 10.1186/s13046-018-0781-8. 29973237 PMC6031179

[34] Cui G , Martin RC , Liu X , Zheng Q , Pandit H , Zhang P , Li W , Li Y . Serological biomarkers associate ultrasound characteristics of steatohepatitis in mice with liver cancer. Nutr Metab (Lond). 2018; 15:71. 10.1186/s12986-018-0304-9. 30323853 PMC6173864

[35] Starley BQ , Calcagno CJ , Harrison SA . Nonalcoholic fatty liver disease and hepatocellular carcinoma: a weighty connection. Hepatology. 2010; 51:1820–1832. 10.1002/hep.23594. 20432259

[36] Knowles BB , Howe CC , Aden DP . Human hepatocellular carcinoma cell lines secrete the major plasma proteins and hepatitis B surface antigen. Science. 1980; 209:497–499. 10.1126/science.6248960. 6248960

[37] Lachenmayer A , Alsinet C , Savic R , Cabellos L , Toffanin S , Hoshida Y , Villanueva A , Minguez B , Newell P , Tsai HW , Barretina J , Thung S , Ward SC , et al. Wnt-pathway activation in two molecular classes of hepatocellular carcinoma and experimental modulation by sorafenib. Clin Cancer Res. 2012; 18:4997–5007. 10.1158/1078-0432.CCR-11-2322. 22811581 PMC3446854

[38] Pandit H , Tinney JP , Li Y , Cui G , Li S , Keller BB , Martin RCG II . Utilizing Contrast-Enhanced Ultrasound Imaging for Evaluating Fatty Liver Disease Progression in Pre-clinical Mouse Models. Ultrasound Med Biol. 2019; 45:549–557. 10.1016/j.ultrasmedbio.2018.10.011. 30527843

[39] Liang W , Menke AL , Driessen A , Koek GH , Lindeman JH , Stoop R , Havekes LM , Kleemann R , van den Hoek AM . Establishment of a general NAFLD scoring system for rodent models and comparison to human liver pathology. PLoS One. 2014; 9:e115922. 10.1371/journal.pone.0115922. 25535951 PMC4275274

[40] Kleiner DE , Brunt EM , Van Natta M , Behling C , Contos MJ , Cummings OW , Ferrell LD , Liu YC , Torbenson MS , Unalp-Arida A , Yeh M , McCullough AJ , Sanyal AJ ; Nonalcoholic Steatohepatitis Clinical Research Network. Design and validation of a histological scoring system for nonalcoholic fatty liver disease. Hepatology. 2005; 41:1313–1321. 10.1002/hep.20701. 15915461

