# Supplementary material for: Carcinogenetic initiation contributed by EpCAM+ cancer cells in orthotopic HCC models of immunocompetent and athymic mice
Source: Oncotarget. 2020 Jun 2;11(22):2047–60. doi: 10.18632/oncotarget.27454 (PMC7275786; doi:10.18632/oncotarget.27454)
Supplement: Supplementary file 1 [file oncotarget-11-2047-s001.pdf]

Carcinogenetic initiation contributed by EpCAM+ cancer cells in orthotopic HCC models of immunocompetent and athymic mice

SUPPLEMENTARY MATERIALS

Routine methods

Protein extraction and immunoblot

To extract total protein, 50–100 mg frozen liver tissues (with or without tumors) were homogenized in RIPA lysis buffer supplemented with protease and phosphatase inhibitors (78443, Thermo scientific, USA). After homogenization in lysis buffer, lysates were transferred to 1.5 mL microfuge tubes and sonicated for 5 seconds on ice. After 30 minutes incubation on ice, lysates were centrifuged for 30 minutes at 14,000 RCF at 4°C. Supernatant containing total cellular protein was collected carefully and transferred to a new labeled 1.5 mL microfuge tube. Purified protein samples were stored at –80°C until further use.

Extracted proteins were quantified using the Bradford assay as per the manufacturer's protocol (500–0006, Biorad, USA) in a 96-well plate format using the ELISA plate reader (MultiSKAN MCC/340, Thermo Fisher Scientific). Equal protein loading was performed (25 µg each, SDS reduced boiled samples) using reducing SDS-PAGE electrophoresis on 10% gel. Protein was then electro transferred to 0.2 micron PVDF membrane (Amersham, GE). Membrane was blocked in 5% non-fat milk or 5% BSA for 1 hour at room temperature. The membrane was then probed for primary antibody overnight at 4ºC, followed by 3x TBST wash for 7 minutes each, and incubated with secondary HRP conjugated antibody for 1 hour at room temperature. Blot was later on washed 3x by TBST, followed by 1 wash of TBS, and then incubated in darkness for 1 minute with chemiluminescence substrate (Santacruz Biotech, Cat # sc-2048). Chemiluminescence signals were recorded using x-ray films or gel documentation system (Biorad XRS+, Biorad, USA). Densitometry analyses were conducted using ImageJ software (NIH, Bethesda, USA).
All antibody source, catalog information, and dilutions are provided in Supplementary Tables 1 and 2.

Supplementary Table 1: Primary antibodies

Target	Source	Catalog	Dilution/assay
β-Catenin	SantaCruz Biotech	sc-7963	1:2000/Western blot
β-Catenin(D10A8)	Cell Signaling	8480	1:100/IHC-P, IF
β-actin	SantaCruz Biotech	sc-81178	1:2000/Western blot
GAPDH	SantaCruz Biotech	sc-365062	1:1000/Western blot
EpCAM (human)	Miltenyi Biotech	130-091-254	1:100/FACS
EpCAM (Mouse)	Miltenyi Biotech	130-102-969	1:100/FACS
EpCAM	Abcam	ab71916	1:100/IHC-P, IF
EpCAM	Abcam	ab68892	1:2000/Western blot
Vimentin	Abcam	ab92547	1:50/IHC
copGFP	Thermofisher	PA5-22688	1:1000/Western blot<br>1:100/IHC
mCherry	Abcam	ab167453	1:1000/Western blot
Ki-67	EMD-Millipore	AB9260	1:100/IHC

Supplementary Table 2: HRP conjugated secondary antibodies

Target	Source	Catalog	Dilution/assay
Anti-Mouse IgG	Cell signaling	7076	1:4000/Western blot
Anti-Mouse IgG	SantaCruz Biotech	sc-2005	1:4000/Western blot
Anti-Rabbit IgG	Cell Signaling	7074	1:5000/Western blot
Anti-Rabbit IgG	SantaCruz Biotech	sc-2004	1:5000/Western blot

Plasma Alanine Transaminase (ALT) and liver Triglyceride (TG) analysis Plasma ALT and TG analysis (cat #) were carried out using commercially available assay kits (Cayman chemicals, Ann Arbor, MI, USA). All assays were performed by following manufacturer's instruction using standards provided in the kit.

Important reagents

Name	Catalog	Source
Hepa1-6	CRL-1830	ATCC
Hep3B	HB-8064	ATCC
HepG2	HB-8065	ATCC
DMEM with 4.5% Glucose	10-013-CV	Corning
FBS	F2442	SIGMA
Antibiotic-antimycotic	30-004-CI	Corning
MEM	11095-080	GIBCO
Non-essential amino acids (100x)	11095-080	GIBCO
Sodium pyruvate (100x)	11360-070	GIBCO
L-Glutamine	G7513	SIGMA
ALT assay kit	700260	Cayman Chemicals
TG assay kit	10010303	Cayman Chemicals

TCGA cohort analysis (408 cases)

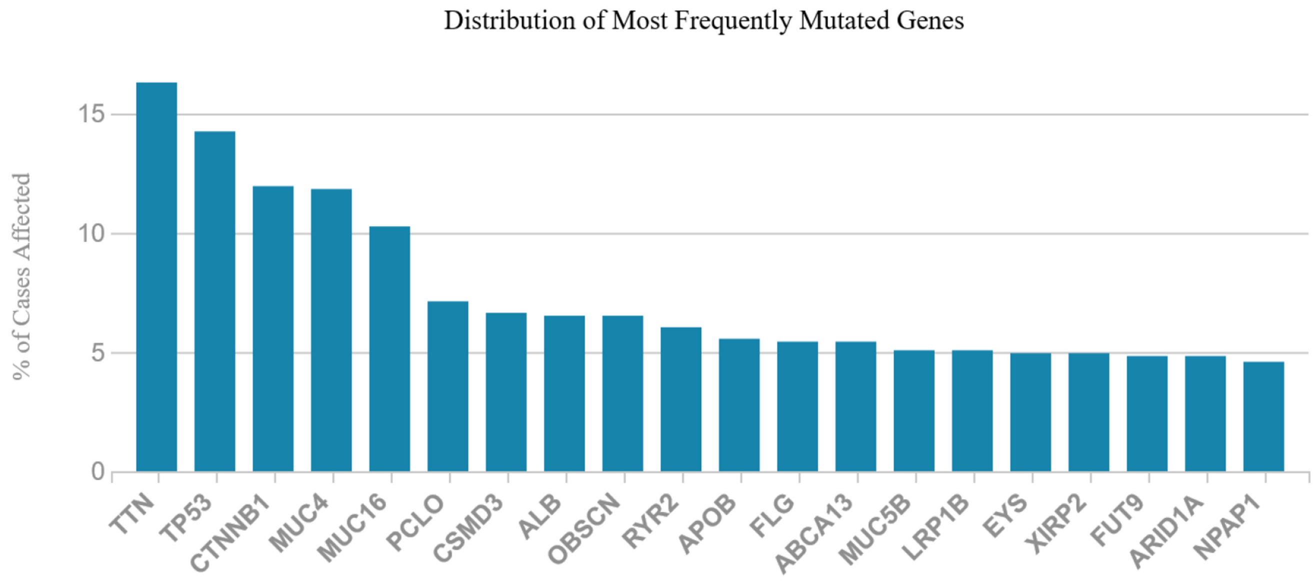

Supplementary Figure 1: TCGA cohort analysis of 408 cases for most frequent mutated genes in liver cancers. Analysis of 408 cases revealed that CTNNB1 (β-catenin) is 3rd most mutated gene in HCC patients. EpCAM is downstream target of CTNNB1, as well as EpCAM is well established CSC marker correlating with poor prognosis in HCC patients. Graph was prepared online from TCGA portal and downloaded "as is". *Last access December 2018.*

Establishing diet induced animal models of
Nonalchoholic fatty liver diseases (NAFLD)

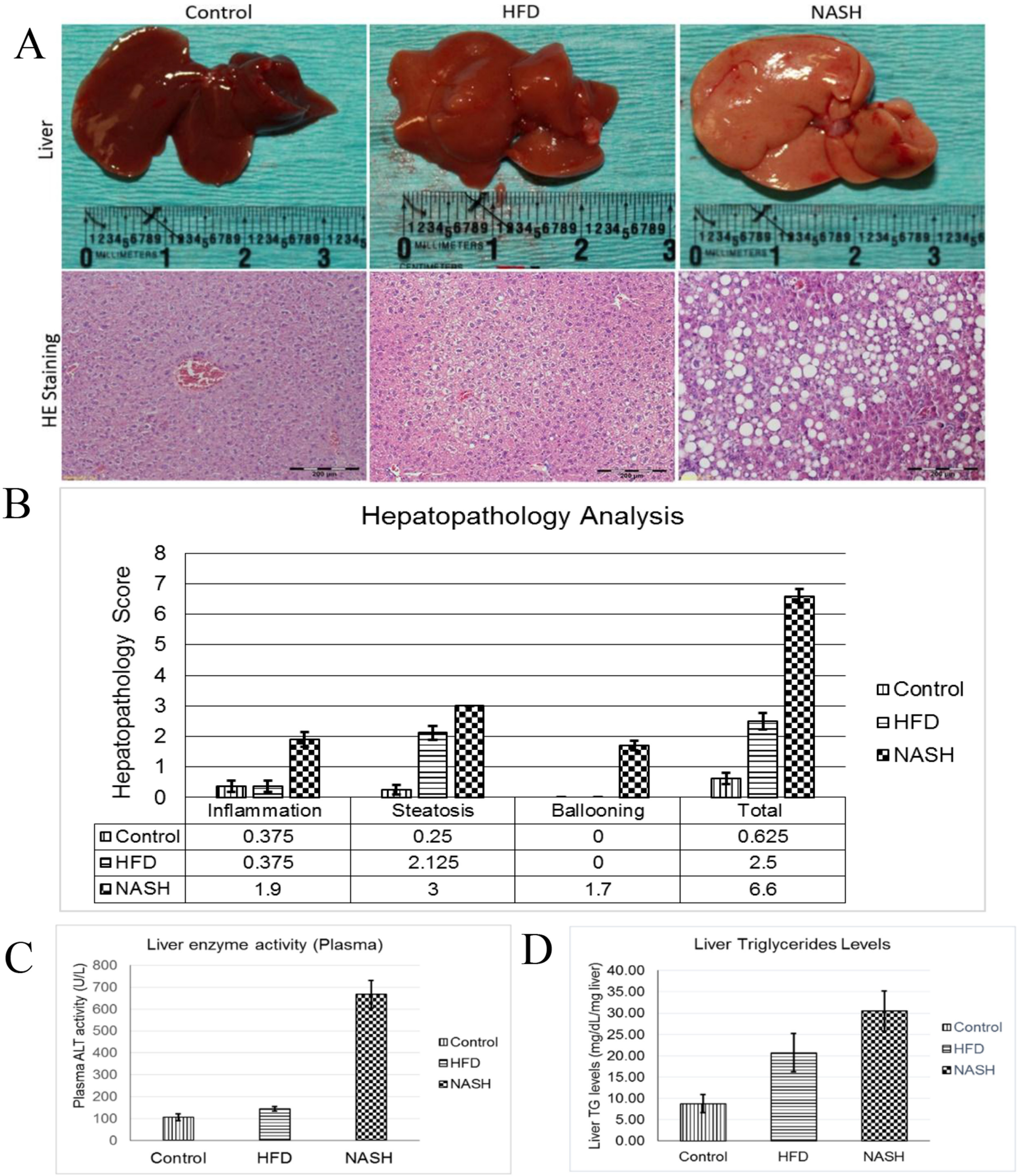

Supplementary Figure 2: Establishing diet induced animal models – liver pathology. Animal experimental groups. (**A**) Representative images of whole liver and corresponding HE staining. 10X magnification (Bar = 200 μm); (**B**) HE staining was analyzed and assign total NAFLD hepatopathology score (0–8), based on inflammation (0–3), steatosis (0–3), and hepatocyte ballooning (0–2) features for each specimen. error bar = SEM, Control: control diet animals (n = 8), HFD: high fat diet animals (n = 8), NASH: CDAFD diet animals (n = 10); (**C**) Plasma ALT activity ($^{\#\#}p < 0.005$, n = 4, error bar = SD); (**D**) Liver triglycerides levels per mg of liver tissue ($^{*}p < 0.05$, n = 4, error bar = SD).

Stable copGFP expressing hepatoma cell lines by lentivirus transduction

A

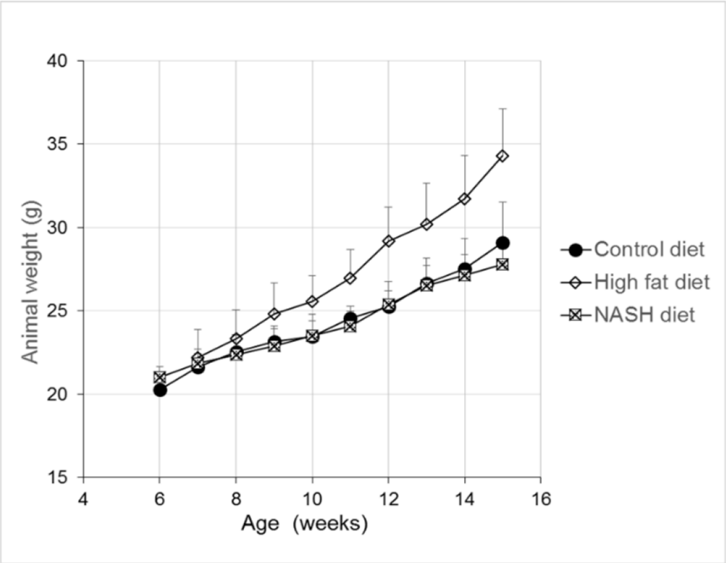

B

Age - 18 weeks	Diet		
	**Control**	**HFD**	**NASH**
**Body weight (gm)**	29.33	34.60	27.40
**S.D.**	2.30	1.66	1.27
**Liver weight (gm)**	1.31	1.84	2.08
**S.D.**	0.09	0.09	0.04
**Liver/body weight ratio(%)**	4.47	4.90	7.63
**S.D.**	0.48	0.34	0.49

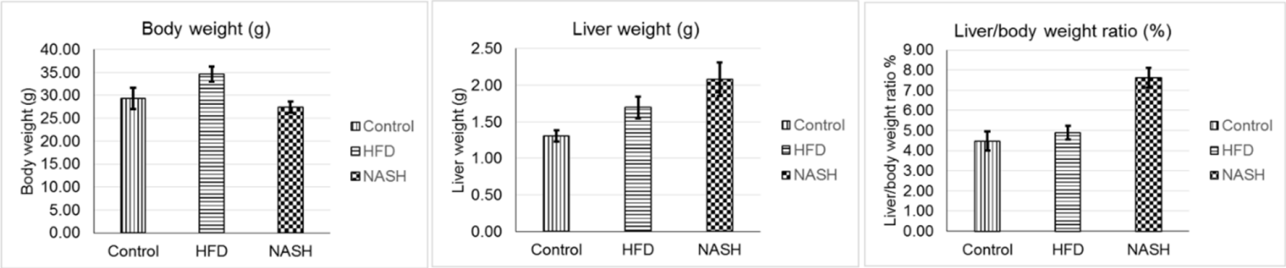

Supplementary Figure 3: Establishing diet induced animal models. Animal were followed for 16 weeks. (**A**) Animal whole body weight changes over the time (error bars = SD); Control diet animals (*n* = 8), High fat diet animals (*n* = 8), NASH diet animals (*n* = 10); (**B**) Animals euthanized at the end of 18 weeks. Comparison table for body weight and liver weight between experimental groups (*n* = 4 in each group, SD = standard deviation).

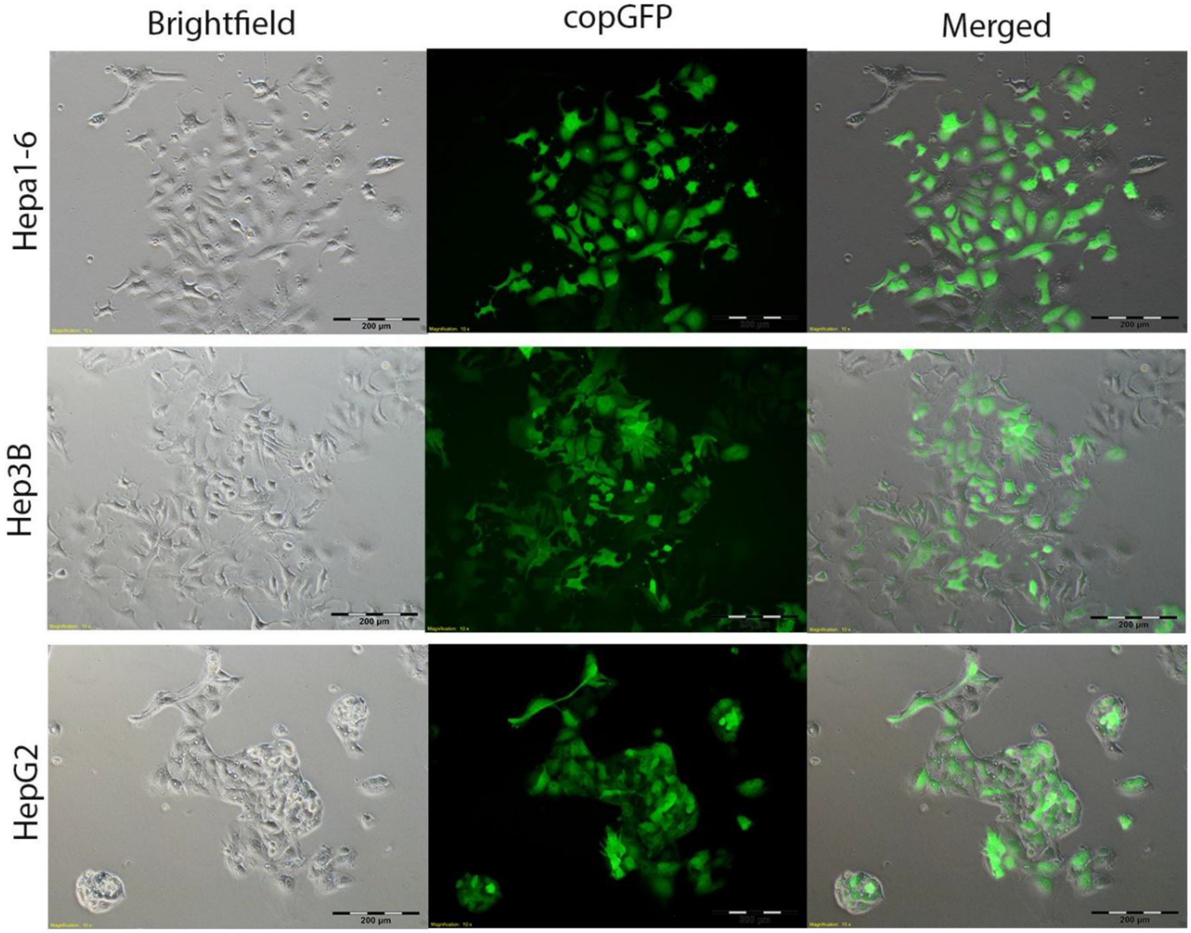

Supplementary Figure 4: Establishing stable copGFP expressing hepatoma cell lines for orthotopic injection. Immunofluorescence images showing copGFP expression in lentivirus transduced hepatoma cell lines in FITC channel. 10X magnification (Bar = 200 µm). Briefly, Hepatoma cell lines (Hepa1-6, HepG2, and Hep3B) were transduced with 6 µg of copGFP-puro-pLenti vector. Clonal selection and expansion performed using complete media with 3 µg/mL puromycine. After 5 generation passage, stable copGFP expressing cells were used for *in vivo* experiments for orthotopic injection.
